# Gendered Social Construction of Adolescent Health Practices Through Digital Detox and Physical Activity

**DOI:** 10.3390/healthcare14010101

**Published:** 2026-01-01

**Authors:** Seungman Lee, Juseok Yun

**Affiliations:** 1 Department of Sport Science, Hankyong National University, Anseong 17579, Republic of Korea; lsm14pe@hknu.ac.kr; 2 Department of Physical Education, Korea University, Seoul 02841, Republic of Korea

**Keywords:** adolescents, digital detox, physical activity, health habits, gender differences, social construction

## Abstract

**Background**: Despite growing concerns about the impact of excessive digital media use on adolescents’ health, few studies have examined how digital detox practices and physical activity interact to influence it, particularly from a gender perspective. **Purpose**: This study investigated the effects of digital detox and physical activity on adolescents’ health habits, focusing on gender differences and sociocultural implications. **Methods**: In February 2025, a self-reported survey was conducted among 652 adolescents (mean age = 15.6, SD = 1.4) residing in Seoul, South Korea, using a quota sampling method. The survey measured four domains: demographic characteristics, digital detox practices, physical activity, and perceptions of health habit improvement. **Results**: Gender-based analyses revealed that female students reported higher engagement in digital detox practices, whereas male students showed greater participation in physical activity and higher levels of health efficacy. Digital detox had a significant positive effect on adolescents’ health habit improvement; however, its effect on physical activity and the effect of physical activity on health habit improvement were not statistically significant. **Conclusions**: These findings suggest that the complex interplay among digital engagement, physical activity, and gender-based social norms shapes adolescents’ health behaviors. To effectively improve adolescent health, strategies should be tailored to address sociocultural dynamics and gender-specific needs and experiences.

## 1. Introduction

Smartphones, computers, and tablets have become essential tools in contemporary daily life [1]. Digital technologies enhance communication, information sharing, and efficiency. However, excessive use of digital devices has been linked to adverse outcomes, such as digital addiction, digital dementia, and digital stress. These risks are particularly concerning for adolescents, who are in a critical stage of psychological and physical development [2,3].

Adolescence is a formative period marked by academic achievement, relationship building, and identity formation. Exposure to a digitally saturated environment shapes adolescents’ lifestyles and health practices [4,5]. Excessive use of smartphones and social media has been associated with reduced attention span, sleep deprivation, lower physical activity levels, and emotional instability, all of which can hinder the development of healthy lifestyle habits [6,7]. Physical activity, in contrast, is recognized as essential not only for physical development but also for emotional well-being, self-esteem, stress relief, and reduced depressive symptoms [8,9]. In response to digital oversaturation, the concept of “digital detox” has emerged, referring to the intentional reduction in or temporary suspension of digital device and social media use to encourage offline activities and restore physical and mental balance [10,11]. Given increasing concerns about excessive screen time negatively affecting adolescents’ mental health, sleep quality, and social interactions, digital detox practices have become particularly relevant in addressing youth health and promoting healthier behavioral patterns.

While digital detox and physical activity have both been identified as important for improving adolescents’ health habits, most studies have examined them in isolation. For instance, Chauhan and Vijayan (2025) [9] reported that digital detox improves academic concentration and emotional regulation, while Li et al. (2024) [12] found that physical activity enhances health awareness and behavioral practices. However, limited empirical research has addressed the interactive effects of these factors or how they jointly influence adolescent health.

Importantly, adolescent health practices are not purely individual choices but are socially constructed within specific sociocultural contexts. Despite this, much existing research treats adolescent health behaviors primarily as psychological or behavioral phenomena at the individual level. Fleming and Agnew-Brune (2015) [13], Dumith et al. (2011) [14], and Stevens and Prinstein (2005) [15] emphasized that gender plays a crucial role in shaping health behaviors through normative expectations, gender roles, and peer dynamics. Similarly, Soh (2024) [16] and Avci, Helwig, and Syed (2024) [17] demonstrated that the intersection of digital environments and social identity influences adolescent health practices. However, studies that examine both the digital and sociocultural dimensions simultaneously remain scarce.

Therefore, this study investigates the effects of digital detox and physical activity on adolescents’ health habits, emphasizing gender differences and sociocultural implications. Patterns of physical activity and digital media engagement among adolescents are influenced by ecological factors (e.g., accessibility to recreational spaces), psychosocial factors (e.g., self-efficacy, peer influences), and prevailing gender norms.

Specifically, the gendered social construction framework suggests that physical activity is often culturally associated with masculinity (e.g., strength, competition, assertiveness), leading male adolescents to exhibit higher participation rates and self-efficacy. Conversely, digital detox practices—which involve self-monitoring, emotional regulation, and attention to mental well-being—may align more closely with societal expectations placed on female adolescents, who are often socialized to prioritize relational health and self-care in ways that manifest as higher awareness of digital media’s negative impact and stronger engagement in self-refrainment behaviors.

By integrating a social/ecological framework, this research examines how digital detox, physical activity, and gender intersect to shape adolescents’ health behaviors. This approach provides a more nuanced understanding of how social structures and digital environments collectively influence youth health, offering theoretical and practical foundations for effective adolescent health promotion strategies.

This study specifically aims to explore improvements in adolescents’ health habits by examining gender differences in digital detox practices and physical activity. It is also expected that the findings will contribute to policy interventions and educational strategies that foster sustainable health behaviors. To achieve this purpose, this study proposes the following hypotheses: first, female adolescents will report higher levels of engagement in digital detox practices, whereas male adolescents will show greater participation in physical activity and more substantial improvements in health habits; second, digital detox will significantly improve adolescents’ health habits; and third, physical activity will significantly contribute to improving adolescents’ health habits.

**Figure 1:**
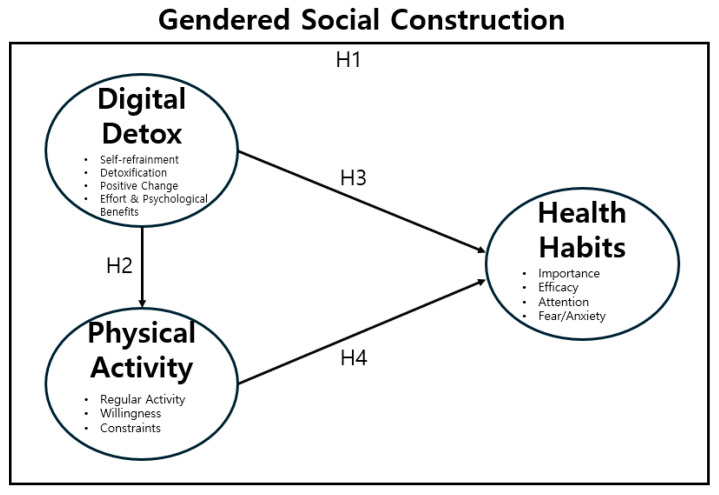
Conceptual framework of this study showing the influence of digital detox and physical activity on adolescent health habits within the context of gendered social construction.

Grounded in the theoretical perspective of gendered social construction, this study conceptualizes digital detox and physical activity as key factors influencing adolescent health. This framework is illustrated in Figure 1.

This study aimed to examine the structural relationships among digital detox, physical activity, and health habits in adolescents, with particular attention to gender differences and sociocultural implications. Based on the literature review and theoretical framework, a research model was developed (Figure 1). The model posits that digital detox influences both physical activity and health habit improvement, and that physical activity, in turn, contributes to enhancing health habits. Furthermore, gender differences were expected in the levels of engagement and behavioral outcomes.

Based on prior research [9,10,11,12,13,14,15] emphasizing the role of gender in health behaviors and the documented differences in engagement with digital media and physical activities according to sex, we first hypothesized gender differences in key variables. Prior research consistently shows that gender plays a crucial role in shaping health behaviors through normative expectations, gender roles, and peer dynamics. Furthermore, digital detox is understood as an intentional effort to restore physical and mental balance, and engaging in self-regulation is known to enhance self-control and motivation for other healthy behaviors, including physical activity. Additionally, both digital detox and physical activity have been independently linked to improvements in overall health awareness, emotional regulation, and self-care practices in adolescents. For instance, digital detox has been reported to improve academic concentration and emotional regulation, while physical activity enhances health awareness and behavioral practices.

Accordingly, the following hypotheses were proposed: (H1) There will be gender differences in digital detox practices, physical activity, and health habits among adolescents. (H2) Digital Detox will have a positive effect on adolescents’ Physical Activity. (H3) Digital Detox will have a positive effect on adolescents’ Health Habit improvement. (H4) Physical Activity will have a positive effect on adolescents’ Health Habit improvement.

## 2. Materials and Methods

### 2.1. Participants

The target population for this study comprised South Korean adolescents enrolled in secondary schools in Seoul. To obtain a balanced distribution across gender and school level, a quota sampling method was employed, targeting 600 students equally divided into four subgroups: middle school boys (*n* = 150), middle school girls (*n* = 150), high school boys (*n* = 150), and high school girls (*n* = 150). To account for potential dropouts or insincere responses, an additional 10% (60 students) was included, resulting in an initial sample size of 660 participants (see Table 1). This 10% buffer was determined based on previous adolescent surveys indicating insincere response rates typically ranging between 8 and 12%. Inconsistent answer patterns or invalid responses were identified as insincere responses and subsequently excluded. Missing data were handled through listwise deletion, excluding questionnaires with more than 20% incomplete responses. Although no formal dropouts occurred, 8 questionnaires were excluded due to insincere responses, yielding a final sample of 652 valid cases for analysis.

However, it should be noted that an a priori power analysis was not conducted; thus, the sample size was primarily determined by practical considerations (e.g., ensuring equal representation for gender and school level) rather than statistical criteria. Crucially, the use of non-probability quota sampling limits the external validity and representativeness of the findings, restricting the generalizability of the results beyond the adolescents surveyed in Seoul. Demographic characteristics collected included gender, school level, parental economic status, and Physical Activity Promotion System (PAPS) grade. The survey was conducted in February 2025. As this study involved minors, informed consent was obtained in advance from both participants and their legal guardians, in compliance with relevant administrative and ethical procedures. This study was conducted in accordance with the principles of the Declaration of Helsinki and was approved by the Institutional Review Board of the Korea National University of Education (protocol code: KNUE IRB-202501-SB-0678-01).

### 2.2. Instruments

The survey consisted of four key components: (1) demographic characteristics; (2) digital detox; (3) physical activity; and (4) improvements in health habits.

First, demographic data included gender (male, female), school level (middle school, high school), perceived parental economic status (within the top 20%, 21–40%, 41–60%, 61–80%, above 81%), and PAPS grade (Grades 1 to 5). Parental economic status was based on participants’ subjective perceptions of their parents’ financial situation. The PAPS grade, widely used in South Korean schools, evaluates physical fitness across five components (power, endurance, muscular strength/endurance, flexibility, body composition) and classifies results into five grades.

Second, due to the absence of a standardized questionnaire for digital detox in the adolescent context, we developed a new scale by adapting and revising items from multiple existing tools. This approach was necessary because existing scales often focus only on specific aspects (e.g., smartphone addiction) and lack the multidimensional focus (self-regulation, well-being gains, emotional effort) required by this study’s conceptual framework. Based on the conceptual framework proposed by Syvertsen et al. (2020) [18], digital detox practices were defined as intentional behaviors aimed at reducing digital dependence and enhancing offline experiences to promote physical and psychological well-being.

Guided by this framework, an initial pool of 22 items was developed and organized into four theoretical dimensions. Following exploratory factor analysis (EFA), 3 items were removed due to low factor loadings (below 0.5), resulting in a final, empirically refined scale of 19 items.

Guided by this framework, the final Digital Detox scale consisted of four dimensions. The first is Self-refrainment (four items), which refers to the intentional and conscious efforts to limit personal digital device usage. The second, Detoxification (five items), indicates the perceived psychological and physical refreshment resulting from reduced use of digital devices. This is complemented by the third dimension, Positive Change (six items), which reflects the anticipated beneficial impacts on daily life quality through reduced digital use. The final item, Effort and Psychological Benefits (four items), describes the psychological gains obtained from deliberate effort, emotional regulation, and sustained engagement in digital detox activities.

Responses were recorded on a 5-point Likert scale (1 = strongly disagree to 5 = strongly agree), and scores were averaged for each subscale, with higher scores indicating greater engagement in digital detox practices. To enhance transparency, Appendix A provides a detailed table identifying the source of each item and specifying whether it was adapted from previous tools or newly developed for this study.

Third, physical activity was measured by adapting items from the Physical Activity Questionnaire for Adolescents (PAQ-A; Martinez-Gomez et al., 2009) [19] and guidelines by Sallis and Saelens (2000) [20], with modifications for age appropriateness and the study context. The final scale comprised 12 items divided into three subdomains: (1) Regular Physical Activity (four items), (2) Willingness to be Physically Active (four items), and (3) Perceived Physical Constraints (four items). Participants rated each item using a 5-point Likert scale (1 = never to 5 = always), with subdomain scores calculated as mean values.

Fourth, improvements in health habits were assessed by adapting items from scales developed by Chen et al. (2011) [21], based on the Health Belief Model, and Jessor et al. (2003) [22], based on Problem Behavior Theory. The final scale consisted of four conceptually distinct subdomains. Perceived Importance of Health (four items) refers to the subjective value and significance adolescents place on their health. Health Efficacy (four items) represents an adolescent’s confidence and belief in their ability to successfully perform health-promoting behaviors. Attention to Health (four items) measures the degree of conscious effort and focus adolescents place on monitoring and actively managing their daily health status. Finally, Fear/Anxiety about Health (four items) describes the level of emotional concern, worry, or fear adolescents experience regarding potential negative health outcomes. Items were measured on a 5-point Likert scale (1 = strongly disagree to 5 = strongly agree), with higher mean scores reflecting more positive or attentive health behaviors. While the overall construct validity was confirmed through EFA, the reliability of the “Fear/Anxiety about Health” subscale was low (α = 0.603); thus, this factor was ultimately excluded from the final path analysis to ensure model rigor and interpretability (as detailed in Section 3.4).

### 2.3. Validity and Reliability of the Survey Instruments

The validity and reliability of the measurement tools were systematically evaluated. First, exploratory factor analysis (EFA) was conducted to verify construct validity for each variable. Although the scales used in this study were adapted from existing research, comprehensive validation of the entire Digital Detox scale had not previously been performed, justifying the use of EFA. The results of the EFA for the digital detox variable are presented in Table 2. Initially, the Digital Detox scale consisted of 22 items adapted and modified from previous studies (e.g., Choi & Kang, 2024; Radtke et al., 2022) [10,23]. Items were revised to better reflect adolescent contexts and study aims (see Appendix A for detailed item sources and modifications). Following the EFA, 3 items (8, 9, and 10) were removed due to factor loadings below 0.5, resulting in a final set of 19 items organized into four factors, accounting for 57.384% of the total variance. These factors were labeled (1) Self-refrainment (four items), (2) Detoxification (five items), (3) Positive Change (six items), and (4) Effort and Psychological Benefits (four items).

The EFA results for the physical activity variable revealed three factors that accounted for a total explained variance of 65.312% (Table 3). All 15 items were retained, as none demonstrated a factor loading below 0.5. The extracted factors were named “Regular Physical Activity” (six items), “Physical Activity Willingness” (five items), and “Physical Constraints” (four items) and were utilized in the subsequent analyses.

The EFA results for the “health habit improvement” variable are presented in Table 4. Four factors were extracted, explaining a total variance of 56.704%. All 20 items were retained, as none demonstrated factor loadings below 0.5. The four extracted factors were identified as “Health Importance” (six items), “Health Efficacy” (four items), “Health Attention” (six items), and “Health Fear and Anxiety” (four items) and were used in the final analysis.

Following this, the reliability of each variable was assessed using Cronbach’s alpha to evaluate the internal consistency of the items. A Cronbach’s α value of 0.70 or higher is generally considered indicative of acceptable reliability; however, values slightly lower (around 0.60 to 0.69) may also be deemed acceptable in exploratory research or for newly developed scales. In this study, reliability coefficients ranged from 0.603 to 0.892, suggesting acceptable to high internal consistency. The subscales with lower reliability (approximately 0.60) were retained, given their exploratory nature, but caution should be exercised when interpreting related findings. The detailed results are presented in Table 5.

### 2.4. Data Analysis Method

To achieve the objectives of this study, data were collected in February 2025 via an online survey (Google Forms; https://forms.google.com) targeting Korean adolescents. The collected responses were analyzed using SPSS (version 18.0; IBM Corp., Armonk, NY, USA). The data analysis procedures were as follows. First, frequency analysis was conducted to examine the demographic characteristics of the participants. Second, EFA was performed to verify the construct validity of the measurement instruments. Third, Cronbach’s alpha was calculated to evaluate the internal consistency and reliability of the variables. Fourth, descriptive statistical analyses (mean, standard deviation, skewness, and kurtosis) were conducted to explore the adolescents’ physical activity levels. Fifth, an independent samples *t*-test was used to examine gender differences across the study variables. Sixth, a correlation analysis was conducted to explore the relationships among digital detox, physical activity, and improvements in health habits. Finally, path analysis was conducted to examine the hypothesized relationships among the key variables. In assessing the model fit, the indices CFI, RMSEA, and SRMR were utilized, following the recommendations of Hu & Bentler (1999) [24]. The significance level for all statistical tests was set at *p* < 0.05.

## 3. Results

### 3.1. Descriptive Statistics of Variables

To examine the descriptive statistics of the variables used in this study, the overall and subfactor scores were analyzed in terms of mean, standard deviation, skewness, and kurtosis. The results are summarized in Table 6. The means ranged from 2.73 to 3.90, and standard deviations ranged from 0.672 to 1.064. Skewness values ranged from ±0.069 to 0.556, and kurtosis values ranged from ±0.102 to 0.596, indicating that the data were normally distributed.

### 3.2. Correlations Among Digital Detox, Physical Activity, and Health Habit Improvement

The results of the correlation analysis among the 11 subvariables related to digital detox, physical activity, and health habit improvement are presented in Table 7. The correlation coefficients ranged from ±0.006 to 0.670. Importantly, none of the variables demonstrated a correlation exceeding 0.8, indicating a “very high correlation”. Therefore, it was concluded that there were no multicollinearity issues, and the data were suitable for multiple regression analysis.

### 3.3. Gender Differences in Digital Detox, Physical Activity, and Health Habit Improvement

#### 3.3.1. Gender Differences in Digital Detox

The hypothesis that “there will be differences in each variable according to gender” was tested for the digital detox variables. The results are presented in Table 8. Among the subvariables, “Positive Change” demonstrated a significant difference, with girls (M = 3.61) scoring higher than boys (M = 3.45) (t = −2.582, *p* = 0.010). However, no significant gender differences were observed for “Self-refrainment” (t = 0.067, *p* = 0.946), “Detoxification” (t = 1.955, *p* = 0.051), and “Effort & Psychological Benefits” (t = 1.783, *p* = 0.075).

#### 3.3.2. Gender Differences in Physical Activity

As depicted in Table 9, the hypothesis that “there would be gender differences in each variable” was partially supported within the domain of physical activity. Specifically, “Regular Physical Activity” was significantly higher among boys (M = 3.38) than girls (M = 2.70) (t =−8.632, *p* < 0.001). Similarly, “Physical Activity Willingness” was also higher among boys (M = 3.00) compared to girls (M = 2.50) (t = −6.520, *p* < 0.001). However, there was no statistically significant gender difference in “Physical Constraints” (t = 1.623, *p* = 0.105).

#### 3.3.3. Gender Differences in Health Habit Improvement

As indicated in Table 10, Hypothesis 1, “there would be gender differences in each variable”, was partially supported in the domain of health habit improvement. Specifically, “Health Efficacy” was significantly higher among boys (M = 3.50) than girls (M = 3.26) (t = −3.524, *p* < 0.001). Similarly, “Health Fear and Anxiety” was also higher among boys (M = 2.95) than girls (M = 2.71) (t = −3.776, *p* < 0.001). However, there were no statistically significant gender differences in “Health Importance” (t = −1.691, *p* = 0.091) or “Health Attention” (t = 1.670, *p* = 0.095).

### 3.4. Path Analysis and Model Verification

To test the hypotheses that (H2) Digital Detox would have a positive effect on Physical Activity, (H3) Digital Detox would have a positive effect on Health Habit improvement, and (H4) Physical Activity would have a positive effect on Health Habit improvement, a path analysis was conducted as shown in Table 11.

The path analysis was conducted to test the structural relationships proposed in the conceptual model. The initial model fit indices were SRMR = 0.071, CFI = 0.852, and RMSEA = 0.130. These indices did not meet the generally accepted criteria for adequate model fit (CFI > 0.90, RMSEA < 0.08, SRMR < 0.08), particularly the CFI and RMSEA values.

Accordingly, the model was refined through post hoc modifications strictly based on statistical criteria to improve the fit. The subfactor “Health Fear & Anxiety” was removed because it exhibited a notably low Squared Multiple Correlation (SMC) value (indicating a lack of reliable variance explanation within the model) and, as previously discussed, a poor reliability coefficient (α = 0.603). This removal was justified based on the inadequate measurement quality and its negative impact on the overall model fit. Following this critical step, covariances were added twice between latent variables with the highest Modification Indices (M.I.) to account for potential shared residual variance not explained by the structural paths.

**Figure 2:**
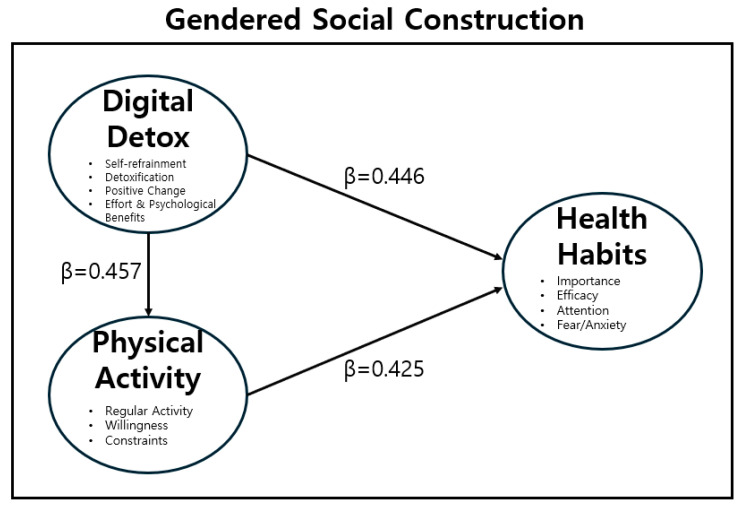
Path coefficients.

After these modifications, the model fit improved substantially to SRMR = 0.059, CFI = 0.929, and RMSEA = 0.097. While the RMSEA remains slightly elevated, the CFI and SRMR values now meet the acceptable criteria for model fit (Hu & Bentler, 1999) [24] (Figure 2).

Examining the path coefficients of the modified model (Table 11), Digital Detox had a significant positive effect on Health Habits (β = 0.446, *p* < 0.001), whereas its effect on Physical Activity (β = 0.457, *p* > 0.05) was not statistically significant. However, Physical Activity showed a positive directional influence on Health Habit (β = 0.425), suggesting that although the effect was not significant, it contributed indirectly to the formation of health habits. Among the subfactors of Digital Detox, perceived benefit (β = 0.805), detoxification (β = 0.703), positive change (β = 0.732), and self-refrainment (β = 0.699) were all found to strongly reflect the Digital Detox construct. Similarly, the subfactors of Health Habit—health attention (β = 0.870), health importance (β = 0.572), and health efficacy (β = 0.569)—showed significant factor loadings, supporting the construct validity of the Health Habit variable. Overall, these results suggest that Digital Detox exerts both direct and indirect positive influences on individuals’ health behaviors, particularly in the formation of healthy lifestyle habits.

## 4. Discussion

This study examined gender differences in digital detox and physical activity to explore ways of improving health habits among adolescents and to provide foundational data for designing effective health promotion programs. To achieve this, an online survey was conducted in February 2025 with 652 Korean adolescents. Based on the results derived from this process, the discussion is presented in two parts: first, an interpretation of the findings, and second, the practical implications of this study.

### 4.1. Interpretation of the Findings

This study analyzed gender differences across three domains—digital detox, physical activity, and health habit improvement—and found results that both align with prior research and offer new insights.

First, in the domain of digital detox, female students scored significantly higher on the “Positive Change” factor than their male counterparts. This result suggests that female adolescents may be more attuned to the negative effects of digital device use and more inclined to engage in self-regulation and detox practices. This finding supports Salepaki et al. (2025) [25], who reported that although women experienced greater psychological resistance to distancing themselves from mobile devices, they also demonstrated heightened awareness of the need for digital detoxification. In essence, female students appeared more likely to practice detox behaviors through self-discipline and reflexivity in digital environments. However, the absence of gender differences in factors such as “Self-refrainment”, “Detoxification”, and “Effort & Psychological Benefits” points to a possible gap between awareness and actual behavior. This suggests that digital detox is influenced not only by individual willpower but also by broader social factors, including peer culture, academic pressure, and social media identity management.

Second, male students reported significantly higher levels of regular physical activity and willingness to engage in such activities compared to female students. These findings are consistent with previous research [26,27], highlighting how adolescent physical activity levels are shaped by gender-based cultural expectations, school-based opportunities, and body image-related social norms [28]. Lower participation among female adolescents cannot be attributed solely to biological differences; rather, it may reflect the influence of gendered embodiment. For instance, social norms that frame high activity levels as “unfeminine” may diminish female students’ self-efficacy and willingness to participate in physical activity. Thus, gender disparities in adolescent physical activity should be understood within structural contexts—such as the gendered organization of physical education, expectations from peers and teachers, and internalized body image concerns.

Third, in the domain of health habit improvement, male students exhibited higher levels of health efficacy, fear, and anxiety, while no significant gender differences emerged in health importance and attention. This suggests that male adolescents may be more responsive to health-related risks in terms of efficacy and concern, whereas everyday attentiveness and general health awareness appear gender-neutral. However, as previous studies have noted [28,29], gender differences in health behavior vary depending on the specific subdimensions examined. Research on positive health behaviors (e.g., nutrition, exercise, self-care) often finds higher engagement among females, while studies on health risk behaviors (e.g., smoking, drinking) report higher prevalence among males.

These complexities indicate that a single behavioral index cannot capture adolescent health practices and must be interpreted within sociocultural, emotional, and perceptual contexts. This study emphasized that adolescents’ approaches to health in a digital environment are constructed differently according to gender. These differences call for a more nuanced analysis beyond binary gender distinctions, one that considers gender performativity and the positionality of adolescents as social actors. Future research should adopt a multilayered analytical approach, integrating additional contextual factors such as socioeconomic background, community resources, and family environment.

This study also examined the effects of four components of digital detox—“Self-refrainment”, “Detoxification”, “Positive Change”, and “Effort & Psychological Benefits”—on adolescents’ health habit improvement, specifically health importance, health efficacy, health attention, and health-related fear and anxiety. The findings suggest that digital detox is a multidimensional and, at times, conflicting form of social action.

First, “Detoxification” and “Positive Change” positively influenced adolescents’ perception of health importance, while “Effort & Psychological Benefits” had a negative effect. This partially aligns with Chotpitayasunondh & Douglas [30], who found that voluntary digital media regulation enhances self-awareness and attention to health. Successfully limiting digital use may prompt cognitive reflection, leading to a reconfiguration of health values. However, the negative impact of “Effort & Psychological Benefits” aligns with Nasir et al. (2025) [31], who noted that detoxing can cause isolation, anxiety, and fear of missing out, leading to emotional exhaustion. Thus, disconnecting from digital devices may instill fear of losing social ties, weakening or defensively reshaping health perceptions.

Second, “Detoxification” and “Effort & Psychological Benefits” positively influenced adolescents’ health efficacy. This supports Radke et al. (2022) [10], who demonstrated that reducing smartphone use enhances self-control and self-efficacy, reinforcing self-directed health behaviors. Emotional experiences tied to “Effort & Psychological Benefits” also promoted efficacy, suggesting that emotion-driven self-regulation motivates healthy behaviors. This highlights the importance of emotional motivation over mere suppression or instruction in promoting healthy practices. However, “Self-refrainment” and “Positive Change” did not significantly affect efficacy, diverging from Gökçearslan et al. (2016) [32], who linked self-control over smartphone use with self-efficacy. In this study, voluntary restraint alone did not yield a sense of efficacy, suggesting that contextual support and structured routines are more vital than restraint itself.

Third, regarding “Health Attention”, “Self-refrainment”, “Positive Change”, and “Effort and Psychological Benefits” demonstrated positive effects, whereas “Detoxification” had no significant impact. This finding aligns with Coyne & Woodruff (2023) [33], who found that digital detox fostered internal awareness, improved bodily perception, and heightened sensitivity to daily routine changes. The lack of significance for “Detoxification” suggests that structural digital restrictions alone may not be sufficient to shift health awareness. Instead, emotional internalization and self-subjectivation are more effective in motivating adolescent health behaviors than mechanical restriction.

Fourth, regarding fear and anxiety, “Effort & Psychological Benefits” had a positive effect, while “Detoxification” had a negative one. This reflects the ambivalence of digital detox: emotional and psychological gains may raise constructive health anxiety and vigilance, yet disconnection can also provoke excessive threat sensitivity or avoidance. This is consistent with Orben et al.’s (2022) [34] theory of digital exclusion, which argues that disconnecting can deprive adolescents of access to information, health content, and online support, thereby amplifying negative health anxieties. In summary, digital detox can generate dual psychological states in adolescents who are deeply embedded in digital connection and information networks. It functions at the intersection of self-discipline and self-care in youth health practices. While this study’s findings broadly align with the existing literature, they also highlight the emotional tensions and negative reactions that may arise during detox. Therefore, future digital detox programs should foster autonomy, provide emotional support, and help adolescents restore social connections.

It is crucial to address the underlying debate regarding the application of digital detox models to the current generation. For today’s adolescents, digital media serve as an essential, non-negotiable tool for leisure, academic achievement, and social identity formation. Consequently, advocating for a reduction in screen time “at any cost” may be unrealistic and potentially harmful, as it risks imposing a lifestyle standard from previous, digitally unconnected generations onto youth whose environments are fundamentally saturated with technology.

This critical perspective suggests that the focus should shift from blanket reduction to fostering mindful and meaningful digital engagement. Instead of only focusing on the negative impacts of overuse, health promotion strategies must recognize digital media as a potential aid for desirable health behaviors. Specifically, digital platforms can be leveraged as crucial tools for health action, such as accessing reliable health information, using fitness tracker applications for regular physical activity, utilizing stress management apps, or participating in online psychological support groups. This enables adolescents to integrate digital tools into a balanced, self-directed healthy lifestyle.

Although certain predictors showed statistically significant associations with health anxiety, the overall explanatory power (R^2^) of the model for this outcome was notably low. This implies that other critical factors influencing adolescents’ health anxiety were not considered in the current model. Hence, caution should be exercised when interpreting these findings, and the observed effects should be considered statistically significant but small in substantive magnitude. Future studies should aim to identify and incorporate additional variables to enhance the explanatory capacity of the models. One possible reason for the low explanatory power is the multidimensional nature of health anxiety, which may not be sufficiently captured by behavioral variables such as digital detox or physical activity alone. For example, emotional regulation difficulties, underlying mental health issues, or social stressors (e.g., academic competition, peer surveillance, family instability) may play a larger role but were not included in this study’s framework. In addition, some predictors may have demonstrated weak or inconsistent effects due to their conditional influence. For instance, voluntary digital detox may enhance health perception in structured settings but fail to do so in socially disconnected environments, thereby diluting its average effect. Likewise, the impact of physical constraints may differ across individuals depending on how they interpret or internalize these experiences. These findings suggest the need for more contextualized models that incorporate psychological, environmental, and relational factors to better understand adolescent health anxiety. Future studies should consider integrating variables such as emotional well-being, perceived social support, and stress-coping strategies to enhance explanatory capacity.

Finally, this study analyzed how the three factors of physical activity—regular physical activity, willingness to engage, and physical constraints—affected adolescents’ health habit improvement. The results partially align with previous studies while revealing some noteworthy distinctions. Only willingness significantly influenced adolescents’ perception of health importance; actual behavior (regular activity) and constraints had no significant effect. This supports Dishman et al. (2004) [35], who emphasized the centrality of intentional factors in shaping adolescent health values and autonomy. Adolescents appear to recognize the importance of health more through internal motivation and meaning-making than through the frequency of their activities, aligning with Ryan & Deci’s (2000) [36] Self-Determination Theory, which posits that internal motivation and value internalization—rather than external performance—more strongly influence sustained engagement and attitudes toward health. In short, “Willingness to Act” serves as a key psychosocial resource that shapes perceptions of health importance, outweighing actual participation or external barriers.

Regarding health efficacy, both regular activity and willingness had positive effects, while constraints had a negative effect. These findings support Bandura’s (1997) [37] Self-Efficacy Theory, which highlights enactive mastery—the actual performance of behavior—as fundamental to developing self-efficacy. Engaging in physical activity directly fosters a sense of control and self-management of health behaviors. Conversely, the negative impact of physical constraints is consistent with Sallis et al. (2000) [20], who identified structural barriers, such as a lack of time, space, and social support, as major deterrents to adolescent activity. These obstacles can undermine self-efficacy, reinforcing a structuralist view that highlights how social and environmental contexts shape health behaviors beyond individual intent.

In terms of health attention, all three factors—regular activity, willingness, and constraints—had positive effects, with willingness being the most influential. Interestingly, constraints also had a positive impact, partially diverging from prior research but aligning with Bélanger et al. (2011) [38], who found a link between adolescent physical activity and health awareness, information-seeking, and self-monitoring. The unexpected positive role of constraints suggests that barriers may provoke reflection on one’s health, increasing awareness. Adolescents who are restricted from engaging in physical activity may become more conscious of their health status. This interpretation aligns with a social constructionist view, which sees health practices not simply as behaviors but as being shaped by social interactions and discourses around opportunity and limitation.

The most striking finding was that, for health-related fear and anxiety, only “Physical Constraints” had a significant positive effect; neither regular activity nor willingness demonstrated influence. This aligns with prior studies illustrating that negative experiences or limitations more strongly impact health-related anxiety. For example, Weems et al. (2010) [39] found that adolescents with physical limitations displayed heightened health sensitivity and anxiety. Restrictions or experiences of being unwell can trigger emotional responses and awareness of health risks, echoing Beck’s (1992) [40] Risk Society Theory, which frames health anxiety as socially constructed. In this light, high activity levels do not necessarily reduce anxiety; instead, constraints and interruptions may heighten awareness of health vulnerability and loss, intensifying anxiety.

In summary, this study revealed that adolescents’ health habits are shaped more by their perception, enactment, and interpretation of experiences—including constraints—than by the frequency of physical activity. Physical activity emerges not as a mere behavioral act but as a meaning-rich social practice intertwined with identity, emotion, and social context. Therefore, adolescent health promotion policies should move beyond encouraging activity alone. They should foster internal motivation, offer psychological support for overcoming constraints, and address structural barriers simultaneously.

### 4.2. Practical Implications of This Study

This study conducted a multilayered analysis of how adolescents’ digital detox practices and physical activity influenced health habit improvement, considering both gender- and factor-based differences. It offers the following practical implications.

First, the findings highlight the need for gender-sensitive health education and intervention strategies. Female students showed greater sensitivity to the negative effects of digital device use and a stronger awareness of the need for detoxification, while male students demonstrated higher participation and willingness in physical activity. These results suggest that adolescent health behaviors are shaped within gendered social contexts and require tailored interventions reflecting sex-specific motivations, constraints, and perceptions of health education and digital detox programs.

Second, the ambivalent effects of digital detox on health habits must be acknowledged. While factors such as “Detoxification” and “Positive Change” positively influenced health awareness and efficacy, “Effort & Psychological Benefits” negatively impacted perceptions due to emotional exhaustion, implying that digital detox strategies should not rely solely on restriction but must include emotional support and peer-based solidarity. Crucially, schools and communities should move beyond punitive restriction programs and instead implement comprehensive digital literacy education and health-oriented media utilization programs. These programs should teach mindful digital engagement, emphasizing how to leverage digital tools (e.g., fitness trackers, health apps) as aids to promote positive physical activity and mental wellness. This approach promotes autonomy, stimulates intrinsic motivation, and fosters collective engagement.

Third, this study confirms that the quality of physical activity experiences and willingness to participate are central to improving adolescents’ health habits. Notably, willingness had a stronger impact than frequency of activity, indicating that internal motivation and personal meaning are more influential than mere participation. Consequently, school physical education should prioritize self-determination and student choice by offering participatory, experiential activities that empower adolescents as active agents in their health practices.

Fourth, the ambivalent role of perceived constraints should be recognized, with support offered to help adolescents transform them into proactive practices. While physical constraints may lower self-efficacy and increase anxiety, they can also enhance self-reflection and health awareness. Thus, rather than promoting exercise alone, schools should implement psychological support, improve environmental conditions, and adopt socially responsive physical education approaches for students facing limitations.

Fifth, the findings provide a crucial basis for developing data-driven, customized intervention strategies tailored according to gender and behavioral factors. Given that digital detox and physical activity were found to operate independently (i.e., the path between them was not significant), policy efforts should focus on separate, targeted support. Specifically, for female adolescents, programs should address the gap between their high awareness of the need for digital detox and their actual practice; strategies must focus on group-based detox activities that provide social and emotional support to mitigate the fear of missing out (FoMO) and isolation, while promoting autonomy in self-regulation. Conversely, for male adolescents, whose engagement in physical activity is higher, interventions should aim to consolidate this activity into greater health efficacy; this can be achieved through achievement-based activities and structured performance goals that reinforce their sense of control over their health behaviors.

This study examined how digital detox and physical activity influence adolescents’ health habits, with attention to gender differences and sociocultural contexts. While the findings are meaningful, several limitations should be noted, and future research directions are suggested accordingly. First, the use of quota sampling without an a priori power analysis limits the representativeness and generalizability of the findings. Future studies should adopt probability sampling and a more robust sample design to improve statistical validity. Second, some regression models showed relatively low explanatory power, suggesting the influence of unmeasured variables. Future research should incorporate key factors, including baseline health status, health literacy, and prior participation in sports, and employ advanced methods such as structural equation modeling to enhance understanding. Third, some items of the Digital Detox scale were modified or newly developed. Additional validation studies are needed to ensure the reliability and construct validity of the measurement tool across diverse populations.

## 5. Conclusions

This study examined the effects of digital detox and physical activity on adolescents’ health habits, with a focus on gender differences and sociocultural implications. The path analysis results revealed that digital detox had a significant and substantial positive effect on health habit improvement (β = 0.446, *p* < 0.001), whereas its effect on physical activity was not statistically significant. The large path coefficient of β = 0.446 suggests that digital detox is a strong predictor of positive health habit formation. Likewise, physical activity showed a positive but non-significant relationship with health habit improvement, suggesting that adolescents’ engagement in physical activity alone may not directly translate into healthier behavioral patterns.

These findings indicate that digital detox practices play a more critical and predictive role in shaping adolescents’ health awareness and lifestyle improvement than physical activity per se. This may reflect the increasing dominance of digital media in adolescents’ daily lives and the psychological relief gained from intentional disconnection. However, the lack of significant associations between digital detox and physical activity suggests that these two behaviors may operate independently rather than interactively in influencing adolescent health.

Gender-based analyses further showed that female adolescents reported higher engagement in digital detox, while male adolescents were more physically active and exhibited greater health efficacy. These patterns highlight the influence of gender norms and sociocultural expectations on adolescents’ health behaviors, underscoring the need for gender-sensitive health promotion strategies.

It is important to acknowledge that the findings of this study, despite their statistical significance, are subject to limitations regarding generalizability, primarily due to the non-probability quota sampling method used and the lack of an a priori power analysis.

In conclusion, fostering adolescents’ healthy lifestyles requires an integrated approach that promotes balanced digital use, emotional regulation, and sustainable health awareness. Schools and policymakers should design programs that encourage mindful digital engagement while also supporting opportunities for enjoyable and inclusive physical activities. Furthermore, educational interventions should reflect gender-specific experiences and motivations, ensuring that both boys and girls can develop positive, self-directed health habits in the digital age.

## Figures and Tables

**Table 1:** Demographic characteristics of the participants.

Variables	Category	*n*	%
Gender	Male	300	46.0
	Female	352	54.0
School Level	Middle School	311	47.7
	High School	341	52.3
Perceived Parental Economic Status	Within Top 20%	110	16.9
	21~40%	141	21.6
	41~60%	310	47.5
	61~80%	62	9.5
	Above 81%	29	4.4
PAPS Grade	Grade 1	124	19.0
	Grade 2	226	34.7
	Grade 3	224	34.4
	Grade 4	68	10.4
	Grade 5	10	1.5
Total		652	100

Tested with frequency analysis.

**Table 2:** EFA of digital detox.

Variable	Item	Factor			
		1	2	3	4
Positive Change	DD12	0.776	0.101	−0.073	0.143
	DD14	0.694	0.070	0.150	0.146
	DD11	0.684	0.150	0.160	0.230
	DD16	0.663	0.199	0.409	−0.002
	DD13	0.646	0.260	0.276	0.200
	DD15	0.616	0.243	0.330	0.154
Detoxification	DD7	0.176	0.747	0.165	0.097
	DD5	0.059	0.728	0.194	0.254
	DD19	0.230	0.664	0.248	0.115
	DD6	0.080	0.581	−0.001	0.295
	DD20	0.430	0.561	0.064	0.162
	DD8	0.150	0.423	0.298	0.355
Effort and Psychological Benefits	DD21	0.175	0.190	0.747	0.281
	DD22	0.048	−0.073	0.657	0.365
	DD17	0.436	0.255	0.655	0.011
	DD18	0.311	0.405	0.631	0.071
	DD10	0.210	0.330	0.448	0.357
Self-refrainment	DD3	0.106	0.141	0.439	0.680
	DD2	0.072	0.215	0.243	0.669
	DD4	0.228	0.363	0.027	0.613
	DD1	0.307	0.148	0.254	0.611
	DD9	0.255	0.355	−0.037	0.451
Eigenvalue		3.681	3.290	2.962	2.691
Variance (%)		16.733	14.956	13.464	12.232
Cumulative Variance (%)		16.733	31.689	45.152	57.384

Tested with EFA.

**Table 3:** EFA of physical activity.

Variable	Item	Factor		
		1	2	3
Regular Physical Activity	PA4	0.827	0.132	−0.037
	PA2	0.817	0.317	0.018
	PA3	0.783	0.073	0.014
	PA1	0.758	0.268	0.010
	PA5	0.694	0.325	0.002
	PA6	0.668	0.447	−0.028
Physical Activity Willingness	PA10	0.257	0.848	0.069
	PA9	0.214	0.845	0.044
	PA11	0.238	0.814	−0.058
	PA7	0.482	0.533	0.201
	PA8	0.473	0.512	−0.117
Physical Constraints	PA13	0.076	0.110	0.846
	PA12	0.059	0.095	0.791
	PA14	0.009	−0.065	0.782
	PA15	−0.139	−0.068	0.696
Eigenvalue		4.120	3.173	2.504
Variance (%)		27.470	21.151	16.692
Cumulative Variance (%)		27.470	48.620	65.312

Tested with EFA.

**Table 4:** EFA of health habit improvement.

Variable	Item	Factor			
		1	2	3	4
Health Importance	HB8	0.737	0.111	0.107	0.042
	HB6	0.736	0.072	0.250	0.030
	HB16	0.724	0.331	0.046	0.072
	HB12	0.724	0.268	0.058	0.036
	HB3	0.669	0.031	0.280	−0.068
	HB1	0.643	−0.041	0.119	−0.022
Health Efficacy	HB13	0.189	0.858	0.151	−0.060
	HB9	0.042	0.766	0.144	−0.032
	HB14	0.197	0.761	0.197	−0.050
	HB15	0.108	0.757	0.205	−0.213
Health Attention	HB7	−0.204	0.128	0.722	0.115
	HB2	0.181	0.016	0.710	−0.002
	HB5	0.338	0.253	0.662	0.054
	HB4	0.397	0.224	0.593	0.010
	HB11	0.196	0.201	0.593	0.106
	HB10	0.262	0.134	0.565	0.158
Health Fear and Anxiety	HB17	0.103	−0.133	−0.011	0.765
	HB19	−0.107	−0.317	0.070	0.713
	HB18	−0.172	0.049	0.210	0.713
	HB20	0.128	0.021	0.049	0.443
Eigenvalue		3.603	2.987	2.833	1.918
Variance (%)		18.017	14.935	14.164	9.588
Cumulative Variance (%)		18.017	32.952	47.116	56.704

Tested with EFA.

**Table 5:** Reliability analysis.

Variable		Cronbach’s α
Digital Detox	Self-refrainment	0.779
	Detoxification	0.790
	Positive Change	0.848
	Effort and Psychological Benefits	0.796
Physical Activity	Regular Physical Activity	0.892
	Physical Activity Willingness	0.853
	Physical Constraints	0.787
Health Habit Improvement	Health Importance	0.833
	Health Efficacy	0.848
	Health Attention	0.788
	Health Fear and Anxiety	0.603

Tested with Internal Consistency Reliability Analysis.

**Table 6:** Descriptive statistics analysis.

Variable		M	SD	Skewness	Kurtosis
Digital Detox	Self-refrainment	2.97	0.907	0.279	−0.116
	Detoxification	3.51	0.792	−0.090	−0.197
	Positive Change	3.54	0.775	−0.187	0.206
	Effort and Psychological Benefits	2.88	0.888	0.405	0.292
Physical Activity	Regular Physical Activity	3.01	1.064	0.097	−0.596
	Physical Activity Willingness	2.73	1.020	0.321	−0.369
	Physical Constraints	2.39	0.911	0.556	0.251
Health Habit Improvement	Health Importance	3.90	0.672	−0.376	0.102
	Health Efficacy	3.37	0.879	−0.069	−0.216
	Health Attention	3.04	0.757	0.202	0.558
	Health Fear and Anxiety	2.82	0.800	0.183	0.184

Tested with Descriptive Statistical Analysis.

**Table 7:** Correlations among variables.

	A	B	C	D	E	F	G	H	I	J	K
Self-refrainment	1										
Detoxification	0.570	1									
Positive Change	0.506	0.533	1								
Effort and Psychological Benefits	0.579	0.512	0.590	1							
Regular Physical Activity	0.284	0.291	0.239	0.363	1						
Physical Activity Willingness	0.272	0.257	0.274	0.426	0.670	1					
Physical Constraints	0.022	−0.067	−0.042	0.103	0.007	0.060	1				
Health Importance	0.185	0.294	0.453	0.156	0.128	0.187	−0.040	1			
Health Efficacy	0.212	0.307	0.200	0.256	0.431	0.433	−0.155	0.358	1		
Health Attention	0.378	0.310	0.409	0.462	0.407	0.529	0.094	0.467	0.431	1	
Health Fear and Anxiety	0.027	−0.080	0.024	0.105	−0.005	0.048	0.449	0.006	−0.200	0.166	1

Tested with Correlation Analysis. The correlation coefficients are significant at the 0.01 level (two-tailed). A = Self-refrainment, B = Detoxification, C = Positive Change, D = Effort and Psychological Benefits, E = Regular Physical Activity, F = Physical Activity Willingness, G = Physical Constraints, H = Health Importance, I = Health Efficacy, J = Health Attention, K = Health Fear and Anxiety.

**Table 8:** Analysis of Gender Differences in Digital Detox.

Variable	M ± SD		t	*p*
	Boy	Girl		
Self-refrainment	2.97 ± 0.957	2.97 ± 0.863	0.067	0.946
Detoxification	3.58 ± 0.817	3.46 ± 0.767	1.955	0.051
Positive Change	3.45 ± 0.860	3.61 ± 0.688	−2.582	0.010 **
Effort and Psychological Benefits	2.95 ± 0.937	2.83 ± 0.842	1.783	0.075

** *p* < 0.01. Tested using an Independent Variable *t*-test.

**Table 9:** Analysis of Gender differences in physical activity.

Variable	M ± SD		t	*p*
	Boy	Girl		
Regular Physical Activity	3.38 ± 1.020	2.70 ± 1.000	8.632	<0.001 ***
Physical Activity Willingness	3.00 ± 1.048	2.50 ± 0.936	6.520	<0.001 ***
Physical Constraints	2.45 ± 0.940	2.33 ± 0.882	1.623	0.105

*** *p* < 0.001. Tested with an Independent Variable *t*-test.

**Table 10:** Analysis of Gender differences in health habit improvement.

Variable	M ± SD		t	*p*
	Boy	Girl		
Health Importance	3.85 ± 0.698	3.94 ± 0.648	−1.691	0.091
Health Efficacy	3.50 ± 0.849	3.26 ± 0.890	3.524	<0.001 ***
Health Attention	3.10 ± 0.771	3.00 ± 0.742	1.670	0.095
Health Fear and Anxiety	2.95 ± 0.831	2.71 ± 0.756	3.776	<0.001 ***

*** *p* < 0.001. Tested with an Independent Variable *t*-test.

**Table 11:** Results of path analysis.

Hypothesis Path			β	Estimate	S.E.	C.R.	*p*
Physical_Activity	←	Digital_Detox	0.457	0.043	0.034	1.275	0.202
Health_Habit	←	Digital_Detox	0.446	0.28	0.044	6.294	***
Health_Habit	←	Physical_Activity	0.425	2.81	2.225	1.263	0.207
self_refrainment	←	Digital_Detox	0.699	1	-	-	-
benefit	←	Digital_Detox	0.805	1.107	0.078	14.149	***
detoxification	←	Digital_Detox	0.703	0.889	0.071	12.582	***
positive_change	←	Digital_Detox	0.732	0.892	0.067	13.233	***
physical_constraints	←	Physical_Activity	0.068	1	-	-	-
PA_willingness	←	Physical_Activity	0.894	13.35	10.341	1.291	0.197
reg_PA	←	Physical_Activity	0.749	12.914	9.997	1.292	0.196
health_important	←	Health_Habits	0.572	1	-	-	-
health_efficacy	←	Health_Habits	0.569	1.242	0.133	9.36	***
health_attention	←	Health_Habits	0.87	1.665	0.153	10.883	***

*** *p* < 0.001. Tested with path analysis.

## Data Availability

The data presented in this study are available upon request from the authors. The data are not publicly available owing to privacy and ethical restrictions.

## Appendix A. Item Source Table for the Digital Detox Scale

**Subscale**	**Source**
Self-refrainment	Adapted from Syvertsen et al. (2020) [18]
Self-refrainment	New (developed by the authors)
Detoxification	Adapted from Syvertsen et al. (2020) [18]
Positive Change	New (developed by the authors)
Effort and Psychological Benefits	Adapted from Syvertsen et al. (2020) [18]

## References

[1] Allcott H., Gentzkow M., Song L. Digital Addiction. NBER Working Paper 2021, No. w28936. https://ssrn.com/abstract=3870938.

[2] Odgers C.L., Jensen M.R. (2020). Annual Research Review: Adolescent mental health in the digital age: Facts, fears, and future directions. J. Child Psychol. Psychiatry.

[3] Ali Z., Janarthanan J., Mohan P. (2024). Understanding digital dementia and cognitive impact in the current era of the internet: A review. Cureus.

[4] Seiffge-Krenke I. (2019). Adolescents’ Health: A Developmental Perspective.

[5] Dienlin T., Johannes N. (2020). The impact of digital technology use on adolescent well-being. Dialogues Clin. Neurosci..

[6] Kaewpradit K., Ngamchaliew P., Buathong N. (2025). Digital screen time usage, prevalence of excessive digital screen time, and its association with mental health, sleep quality, and academic performance among Southern University students. Front. Psychiatry.

[7] Hancox R.J., Milne B.J., Poulton R. (2004). Association between child and adolescent television viewing and adult health: A longitudinal birth cohort study. Lancet.

[8] Janssen I., LeBlanc A.G. (2010). Systematic review of the health benefits of physical activity and fitness in school-aged children and youth. Int. J. Behav. Nutr. Phys. Act..

[9] Chauhan A., Vijayan D. (2025). The relationship between digital detox, emotional regulation, and productivity among young adults. World J. Adv. Res. Rev..

[10] Radtke T., Apel T., Schenkel K., Keller J., von Lindern E. (2022). Digital detox: An effective solution in the smartphone era? A systematic literature review. Mob. Media Commun..

[11] Mohamed S.M., Abdallah L.S., Ali F.N.K. (2023). Effect of digital detox program on electronic screen syndrome among preparatory school students. Nurs. Open.

[12] Li N., Wang D., Zhao X., Li Z., Zhang L. (2024). The association between physical exercise behavior and psychological resilience of teenagers: An examination of the chain mediating effect. Sci. Rep..

[13] Fleming P.J., Agnew-Brune C. (2015). Current trends in the study of gender norms and health behaviors. Curr. Opin. Psychol..

[14] Dumith S.C., Hallal P.C., Reis R.S., Kohl H.W. (2011). Worldwide prevalence of physical inactivity and its association with human development index in adolescents. Prev. Med..

[15] Stevens E.A., Prinstein M.J. (2005). Peer contagion of depressogenic attributional styles among adolescents: A longitudinal study. J. Abnorm. Child Psychol..

[16] Soh Y. (2024). A framework for investigating the relationship between digital environments and identity development. Soc. Personal. Psychol. Compass.

[17] Avci S., Helwig C.C., Syed M. (2024). A systematic review of social media use and adolescent identity development. Adolesc. Res. Rev..

[18] Syvertsen T., Enli G. (2020). Digital detox: Media resistance and the promise of authenticity. Convergence.

[19] Martinez-Gomez D., Martinez-de-Haro V., Pozo T., Welk G.J., Villagra A., Calle M.E., Veiga O.L. (2009). Reliability and validity of the PAQ-A questionnaire to assess physical activity in Spanish adolescents. Rev. Esp. Salud Publica.

[20] Sallis J.F., Saelens B.E. (2000). Assessment of physical activity by self-report: Status, limitations, and future directions. Res. Q. Exerc. Sport.

[21] Chen M.F., Wang R.H., Schneider J.K., Tsai C.T., Jiang D.D., Hung M.N., Lin L.J. (2011). Using the Health Belief Model to understand caregiver factors influencing childhood influenza vaccinations. J. Community Health Nurs..

[22] Jessor R., Turbin M.S., Costa F.M., Dong Q., Zhang H., Wang C. (2003). Adolescent problem behavior in China and the United States: A cross-national study of psychosocial protective factors. J. Res. Adolesc..

[23] Choi N.-Y., Kang H.-W. (2024). A Study on the Effectiveness of Digital Detox Applying a Therapy Recreation Program for Adolescents. J. Leis. Stud..

[24] Hu L.-T., Bentler P.M. (1999). Cutoff Criteria for Fit Indexes in Covariance Structure Analysis: Conventional Criteria versus New Alternatives. Struct. Equ. Model..

[25] Salepaki A., Zerva A., Kourkouridis D., Angelou I. (2025). Unplugging youth: Mobile phone addiction, social impact, and the call for digital detox. Psychiatry Int..

[26] Twenge J.M., Martin G.N., Spitzberg B.H. (2020). Gender differences in associations between digital media use and psychological well-being: Evidence from three large datasets. J. Adolesc..

[27] Steene-Johannessen J., Hansen B.H., Dalene K.E., Anderssen S.A. (2020). Variations in accelerometry measured physical activity and sedentary time across Europe—Harmonized analyses of 47,497 children and adolescents. Int. J. Behav. Nutr. Phys. Act..

[28] Guinhouya B.C., Samouda H., de Beaufort C. (2013). Level of physical activity among children and adolescents in Europe: A review of physical activity assessed objectively by accelerometry. Public Health.

[29] Kim H., Park K.H., Park S. (2021). Gender differences in sexual behaviors and their relevance to mental health among high school students with sexual experience in South Korea. Int. J. Environ. Res. Public Health.

[30] Chotpitayasunondh V., Douglas K.M. (2018). The effects of “phubbing” on social interaction. J. Appl. Soc. Psychol..

[31] Nasir P., Gillani S.A., Rashid A. (2025). From perceived ostracism to mental exhaustion: Illuminating the link of doomscrolling and digital detox. Pak. Soc. Sci. Rev..

[32] Gökçearslan Ş., Mumcu F.K., Haşlaman T., Çevik Y.D. (2016). Modelling smartphone addiction: The role of smartphone usage, self-regulation, general self-efficacy and cyberloafing in university students. Comput. Hum. Behav..

[33] Coyne P., Woodruff S.J. (2023). Taking a break: The effects of partaking in a two-week social media digital detox on problematic smartphone and social media use, and other health-related outcomes among young adults. Behav. Sci..

[34] Orben A., Tomova L., Blakemore S.-J. (2022). Digital access constraints predict worse mental health among adolescents during COVID-19. Sci. Rep..

[35] Dishman R.K., Motl R.W., Saunders R., Felton G., Ward D.S., Dowda M., Pate R.R. (2004). Self-efficacy partially mediates the effect of a school-based physical-activity intervention among adolescent girls. Prev. Med..

[36] Ryan R.M., Deci E.L. (2000). Self-determination theory and the facilitation of intrinsic motivation, social development, and well-being. Am. Psychol..

[37] Bandura A. (1997). Self-Efficacy: The Exercise of Control.

[38] Bélanger M., Casey M., Cormier M., Filion A.L., Martin G., Aubut S., Chouinard P., Beauchamp J. (2011). Maintenance and decline of physical activity during adolescence: Insights from a qualitative study. Int. J. Behav. Nutr. Phys. Act..

[39] Weems C.F., Silverman W.K., Rapee R.M., Pina A.A. (2003). The role of control in childhood anxiety disorders. Cogn. Ther. Res..

[40] Beck U. (1992). Risk Society: Towards a New Modernity.

